# Bovine Anaplasmosis: Will there ever be an almighty effective vaccine?

**DOI:** 10.3389/fvets.2022.946545

**Published:** 2022-10-05

**Authors:** Elizabeth Salinas-Estrella, Itzel Amaro-Estrada, Mayra E. Cobaxin-Cárdenas, Jesús F. Preciado de la Torre, Sergio D. Rodríguez

**Affiliations:** Unidad de Anaplasmosis, Centro Nacional de Investigaciones Disciplinarias en Salud Animal e Inocuidad, Instituto Nacional de Investigaciones Forestales Agrícolas y Pecuarias (INIFAP), Jiutepec, Morelos, Mexico

**Keywords:** *Anaplasma marginale*, immunity, bovine anaplasmosis, inactivated vaccine, live vaccines

## Abstract

Bovine anaplasmosis is a tick-borne bacterial disease with a worldwide distribution and the cause of severe economic losses in the livestock industry in many countries, including México. In the present work, we first review the elements of the immune response of the bovine, which allows ameliorating the clinical signs while eliminating the majority of the blood forms and generating an immunologic memory such that future confrontations with the pathogen will not end in disease. On the other hand, many vaccine candidates have been evaluated for the control of bovine anaplasmosis yet without no commercial worldwide effective vaccine. Lastly, the diversity of the pathogen and how this diversity has impaired the many efforts to control the disease are reviewed.

## Introduction

Bovine anaplasmosis is a tick-borne rickettsial disease with worldwide distribution. It causes severe economic losses in the livestock industry in many countries, including México (1, 2). *Anaplasma marginale*, the causative agent, is a vector-borne, Gram-negative bacterium that replicates in mature erythrocytes of cattle and other ruminants (3). The disease is more notorious in cattle older than 2 years old and is rarely apparent in younger animals (1). Clinical signs include anorexia, jaundice, abortion, weight loss, decreased meat and milk production, and, potentially, death (2). Losses due to bovine anaplasmosis reach billions of dollars worldwide (4).

Cattle affected with any clinical form of anaplasmosis may recover when antibiotics are administered promptly. Yet, the pathogen may not be completely eliminated; thus, some cattle may be lifelong carriers (5, 6), acting as reservoirs for susceptible or healthy animals under inappropriate veterinary practices (7).

*A. marginale* has a small genome composed roughly of 1.2 mega pair bases, with two gene superfamilies and a highly diverse genetic composition among geographical isolates (8, 9). This genetic composition provides the capability to produce different variants of major surface proteins (MSPs) that help the pathogen evade the immune response and remain within the animal throughout its life span (10).

Ticks are an important *A. marginale* reservoir and maintain the pathogen in nature. While one-host female ticks (e.g., *Rhipicephalus microplus*) do not readily move from one animal to another, their male counterparts do so when cattle are held nearby, enabling transmission from an infected to a non-infected host (11, 12). Furthermore, unfed larvae from infected female *R. microplus* ticks can transmit *A. marginale* when they feed for the first time on a susceptible host (13).

Vaccination is considered the best option to control infectious diseases. For some diseases, there may be several vaccine options for different species, ages, or sexes (14), but bovine anaplasmosis is not such a case. The problem of bovine anaplasmosis is that several factors allow the pathogen to remain in the host for its entire lifetime (1). There is still no practical solution to eradicate the disease because the tick vector would also have to be eradicated. The absence of immunity in a large proportion of the herd in the presence of ixodicide failure can cause disasters in the form of massive outbreaks (15). Conversely, epidemiological stability, which refers to a minimum of carriers of both ticks and *Anaplasma*, may not be practical when new susceptible animals or a new strain is introduced into an infected herd (16).

Here, we first reviewed the protective immune response of bovine, which allows controlling *A. marginale* infections. Then, we reviewed some of the most relevant efforts to develop vaccines, their drawbacks, and their potential for success. Finally, we question the possibility of a vaccine that could protect cattle worldwide, even in the presence of controlled infection.

## Immunology of bovine anaplasmosis

*Anaplasma marginale* enters the vascular system through a vector bite. Initial bodies contact bovine erythrocytes through adhesins present on the outer surface of the bacteria (17) and their (still uncharacterized) cognate receptors on the bovine erythrocyte. This inclusion body releases the initial bodies, which infect other erythrocytes, multiply, and promote further infection. Erythrocytes are not destroyed by penetration or release of the initial bodies from the bacteria (3).

The most accepted model for the protective immune response indicates that IgG2 is necessary for the clearance of *A. marginale* through neutralization of outer membrane B-cell epitopes. At the same time, CD4+ T-cell-mediated macrophage activation is essential for opsonization and microbial killing (18).

Many studies showed that specific antibodies directed to relevant *A. marginale* protein epitopes correlate with the protection against two of the most important clinical indicators of disease: acute rickettsemia and anemia (19). In this model of immunity, antibodies alone are insufficient for protection, but they are required for macrophage opsonophagocytosis (19).

**Figure 1:**
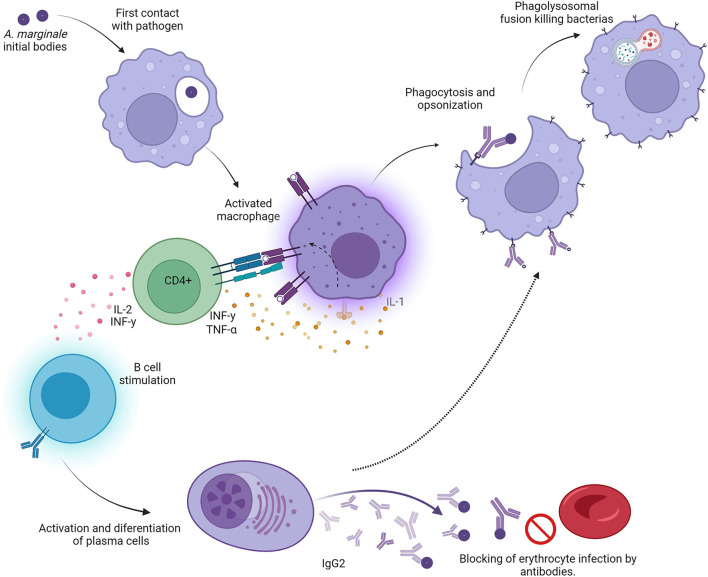
Graphic representation of the “desired” Th1-type immune response in animals protected against bovine anaplasmosis. Initial bodies released from the bovine erythrocyte encounter antigen-presenting cells (macrophages and/or dendritic cells), which engulf, process, and present epitopes to both B and T cells. CD4^+^ T cells are activated when they encounter the appropriate epitopes in the context of class II major histocompatibility receptors on antigen-presenting cells. These activated T cells secrete specific lymphokines, including IFNγ and IL-2. B cells are activated and stimulated by CD4^+^ T-cell cytokines and become IgG2-producing plasma cells. The large quantities of IgG2 can neutralize initial bodies, rendering them incapable of infecting normal erythrocytes. At the same time, activated macrophages destroy opsonized bacteria faster in the presence of IFNγ, leading to recovery from the clinical syndrome.

CD4+ T cells expressing interferon γ (IFN-γ) are central to this model of protective immunity against *A. marginale* (Figure 1). IFN-γ enhances IgG2 production in cattle (20), and bovine CD4+ T cells expressing IFN-γ have been shown to induce IgG2 secretion in B cells. Similarly, IFN-γ activates macrophages to enhance Fc receptor expression, phagocytosis, phagolysosomal fusion, and the production of rickettsiacidal nitric oxide (21). This model provides the basis for specific cell-mediated immunity against a pathogen limited strictly to intraerythrocytic parasitism, which thus cannot be directly targeted by major histocompatibility complex (MHC) class I-restricted cytotoxic lymphocytes.

Immunization with *Anaplasma* outer membrane fractions that have been cross-linked and consist of native proteins induced a protective immune response dependent on Th1 lymphocytes (22, 23). This response is represented by the high production of IgG2, IFN-γ, and IL-2 (21, 24). Unlike IgG1, IgG2 is an opsonizing immunoglobulin; once it binds with its epitope, the immunoglobulin Fc region activates local macrophages and neutrophils, which become more efficient in removing free initial bodies. In turn, the secretion of IFN-γ by specific Th1 lymphocytes augments local phagocyte activity and eliminates opsonized initial bodies [(21, 24); Figure 1].

This model of protective immune response was mostly based on immunization and challenge experiments carried out in calves, mainly 6-month-olds. However, cattle younger than 12 months do not usually develop clinical disease, despite being infected with *A. marginale* (1, 21). This model of protective immunity was validated by inoculating heifers older than 1 year with an inactivated vaccine incorporated with Quil-A saponin. This adjuvant is known to induce a Th1-type immune response in humans (25). In this experiment, the vaccinated animals showed a Th1-type immune response before and after a challenge with a virulent Mexican strain. Most vaccinated animals had higher IgG2 than IgG1 titers, a higher proportion of CD4+ than CD8+ T cells, and produced IFN-γ. Moreover, few animals that presented the inverse proportions of IgG and T cells (IgG2 < IgG1 and CD4+ < CD8+) suffered from acute clinical signs of disease and required treatment to avoid death (26).

Although the mechanisms of CD4+ preference over CD8+ are still unknown, it is clear that the CD4+-lymphocyte immune response is necessary for protection against natural infection or under conditions of artificial immunization (26). Thus, recent studies on potential native or recombinant antigens for vaccine use include CD4+ T-cell epitopes. Here, we reviewed some of the different efforts to produce an effective vaccine that will stimulate the ideal type of immune response and solidly protect cattle against anaplasmosis with the support of other practices.

## Antigenic diversity vs. antigenic variability

Early studies hypothesized great diversity among *A. marginale* strains from different geographical locations. Molecular analyses confirmed this diversity (27); for example, the *Msp1a* gene, encoding for a major surface protein (Msp), includes up to 11 highly similar tandem repeat sequences (28). To date, there are over 700 accession numbers (NCBI) for complete or partial amino acid sequences for this protein alone (29–32). By analyzing isolates from different regions and even among organisms isolated in the same location or same animal, it has been shown that, while some proteins are conserved, others are not (33). Furthermore, proteins encoded by multigene families are even more diverse than proteins encoded by a single gene. Msp1b, for instance, is encoded in at least two complete and three partial genes that recombine to produce variants of the same protein (28, 34). Other major surface proteins like Msp4 or Msp5 are coded in single genes and are highly conserved. Unfortunately, these proteins are not good vaccine candidates; Msp4 immunization does not always produce specific antibodies (35), whereas Msp5 induces large amounts of antibodies that are not protective (36, 37).

Conversely, Msp2 and Msp3 proteins are each encoded in multigene families composed of a main gene and a variable number of (5–7) partial genes. The main gene recombines with each partial gene fully or in segments through a mechanism known as segmental gene conversion. Variants emerge at cycles of 6–8 weeks throughout the host's lifetime (38, 39). This information indicates that none of these proteins are suitable vaccine candidates despite their vital role in erythrocyte infection. Other bacterial proteins, like type 4 secretion system (TFSS) proteins, react with IgG2 *in vivo*; however, they fail to induce protective immunity when inoculated in cattle (40). Future proposals of proteins as vaccine candidates should consider their molecular and antigenic diversities.

## Types of vaccines

### Live vaccines

Live heterologous *A. marginale* vaccines have been used since the early 20^th^ century. Theiler reported the presence of *Anaplasma centrale* as a different organism in South Africa (41), which was less virulent and could be used for the immunization of naive cattle against the more virulent *A. marginale* (42). *A. centrale* is still being produced as a live—very often as trivalent (*Babesia bovis, B. bigemina*, and *A. centrale*)—vaccine in South Africa, Australia, Argentina, Brazil, Uruguay, Israel (43–47), among others. Thousands of doses of these vaccines are distributed every day, enabling more affordable cattle production. However, a few reports identified *A. centrale* as the cause of outbreaks with fatalities (48) or failure to induce immunity against *A. marginale* challenge (49, 50). Potgieter (42), citing Theiler, stated that immunization with *A. centrale* attenuates the clinical presentation of *A. marginale* infection but does not prevent the infection from occurring. Thus, live agents should be used with caution.

Using live virulent *A. marginale* as an immunogen involves the sub-inoculation of blood from infected carriers to susceptible animals paired with treatment—premunition—to avoid acute or fatal anaplasmosis (49–52). In turn, using infected blood from a carrier or a patent animal implies injecting an unknown number of infected erythrocytes; thus, the monitoring period varies from one inoculation to another (53). The results of these types of trials show the unreliability of the method: in some animals, 10 μl of infected blood can induce an infection with a delayed incubation period, while other animals showed no clinical signs (53).

Some virulent strains have been attenuated by passaging organisms in “unnatural” hosts. *A. marginale* has been passaged in splenectomized sheep and deer (53, 54). These organisms were partially successful in several countries, including Mexico, Perú, and Colombia (55–57). The efficacy of virulent, sheep-attenuated, and deer-attenuated vaccines is different; the deer-passaged strain failed to induce solid protection (56), and live virulent and sheep-attenuated strains induced similar protection (53). However, calves administered with virulent strains showed less severity of the signs after an artificial or natural inoculation challenge 6 months later than those previously administered with the sheep-attenuated strain (54). The difference in the presentation of clinical signs in the immunized animals has been attributed to the lack of cross-protection between strains or to the magnitude of the antibody response between the immunizing and the challenge strains (57).

*Anaplasma marginale* strains of naturally low virulence have also been tested as potential vaccines. One of the earliest efforts (53) used the Florida strain. Further efforts using local strains have been reported in Australia, Mexico, Brazil, to mention a few (58–61). All these efforts have one thing in common: fresh or frozen infected erythrocytes were inoculated to induce a mild clinical syndrome (premunition). As occurs when using infected blood, there is always the risk of transferring other blood-borne pathogens, including *Babesia, Ehrlichia*, or virus. For example, in Australia, the bovine leucosis virus (BLV) was transmitted in BLV-free cattle with dire consequences (62).

One of the expected advances in bovine anaplasmosis is the *in vitro* cultivation of the pathogen. This has been explored for a long time in mammalian systems without success. Early reports claimed that the pathogen could be grown in several conditions, including rabbit bone marrow tissue cultures (63), bovine erythrocytes (64, 65), bovine erythrocytes co-cultured with endothelial cells (66), and endothelial cells (67). Most of these efforts have not been replicated by others or even the same authors.

More recently, *A. marginale* has been cultured in several tick cell lines (68, 69). Some of these reports showed that major surface proteins expressed in erythrocytic stages in *A. marginale* and *A. centrale* are also expressed when cultured *in vitro* in tick cell lines (70–72). Immunization of cattle with *in vitro*-cultured *A. marginale* did induce an antibody immune response but not to the expected protection level (68, 71). The protection level was, at best, comparable to immunization with crude antigens derived from initial bodies against a homologous challenge (72). However, tick cell lines are still a tool to isolate *A. marginale* from field outbreaks or even carriers, providing material for further characterization (73, 74).

### Inactivated vaccines

Facing the impossibility of using *A. centrale*, early vaccine efforts used lyophilized blood with high numbers of infected erythrocytes that was reconstituted with an oil adjuvant and inoculated into susceptible hosts (53). Earlier inactivated vaccines were unpractical due to the high content of erythrocyte stroma that was probably related to cases of neonatal isoerythrolysis in calves born to vaccinated dams (4).

Inactivated preparations of purified initial bodies from bovine erythrocytes have also been used. A study immunized adult cattle with the preparation of initial bodies solubilized with detergent and incorporated with Quil-A saponin. After immunization, the animals were challenge-inoculated with 1 × 10^9^ infected erythrocytes of a heterologous strain, but the vaccine was not effective (75). Similar observations were also reported with other inactivated vaccines (76, 77). In an effort to cover a wider antigenic spectrum, a Mexican vaccine essay used three strains that shared major surface proteins Msp1a and Msp4; only the animals (yearlings) challenged with the combination that included the homologous strain resisted the challenge, while all other groups had to receive chemotherapy to prevent death under stall conditions (78). By contrast, a similar preparation [in adjuvant and antigen composition and applied by the same route as in the study mentioned in Rodríguez et al. (78)] was used to immunize yearlings under the same regime as the previous experiment under ranch conditions; the immunized animals produced specific antibodies (as determined by iELISA) against all three strains and were protected against naturally tick-transmitted anaplasmosis a year after a two-dose vaccine application (79). In both of these studies, high antibody titers were observed, regardless of the vaccination settings. Although the results of these two experiments seem contradictory, one major difference was the challenge dose and settings: the first essay used 10^8^ freshly reactivated infected erythrocytes as inoculum under stall conditions, whereas in the second experiment, animals acquired the infection gradually as they were infested by ticks in the ranch where the experiment was carried out.

University Products LLC (Louisiana, USA) currently offers the only inactivated commercial vaccine against bovine anaplasmosis (https://www.prnewswire.com/news/university-products-llc/). The producer claims that, while the vaccine does not prevent infection with virulent *A. marginale*, it induces enough immunity to protect cattle against the clinical syndrome. This vaccine has been used in thousands of animals for over two decades since its development. The methodology for extracting the initial bodies is referred to in the study mentioned in Orozco-Vega et al. (80), McCorkle et al. (81).

### New-generation vaccines

#### DNA and recombinant proteins

The search for immunodominant and subdominant proteins in the published *A. marginale* genomes has yielded lists of potential vaccine candidates, including outer membrane proteins (OMPs), major surface proteins (MSPs), and several type 4 secretion system (TFSS) proteins (82). Early reports explored Msp1a, Msp1b, Msp2, Msp3, Msp4, and Msp5 (83) recombinant proteins for the immunization of calves. Table 1 shows a list of MSPs and other membrane proteins that have been studied as potential candidates for immunization in the form of DNA, plasmids, recombinant proteins, or even synthetic peptides.

**Table 1:** A summary of some of the many vaccine candidates, animal models, the stage of the study and adjuvants used for immunization against bovine anaplasmosis.

**Molecule/*gene***	**Putative function -**	**Feature**	**Localization**	**Stage of study**	**Adjuvant**	**References**
*msp-1α*	Adhesin to red blood cells & tick epithelial cells	R-DNA	MSP	Immunization of mice and/or bovines	DNA	(83–85)
rMSP1a, rMSP1b, rMSP4, and rMSP5	Adhesion, molecular marker	R-proteins	MSP	Immunization of mice and/or bovines	ISCOM/and ISCOMATRIX	(86, 87)
Msp1a	Adhesin to RBC & Tick epithelial cells	R-proteins	MSP	Immunization of mice and/or bovines with nanotubes	Quil-A/SV-100 silica vesicles; Carbon nanotubes/Emulsigen^®^	(88, 89)
				Ear implant & SC inoculation in yearlings	Quil A and Montanide/ISA201	(90)
Msp1b	Adhesin to RBC & Tick epithelial cells	R-proteins	MSP	Characterization/immunization of bovines	Saponin	(26)
*msp1β*		DNA-plasmid			Dextran and Quil-A/ Montanide ISA 61 VG	(91)
Msp2 &/or Msp3	Immune response evasion/antigenic variants	R-proteins	MSP	Characterization/immunization of mice and/or bovines	Saponin	(24)
Msp4	Molecular marker/Unknown function	R-proteins	MSP	Characterization/immunization of rabbits and bovines	ISCOM/ DNA	(87, 92, 93)
Msp5	Immune response evasion	Plasmid-Vectored/recombinant Prime-booster-vaccine	MSP	Characterization/immunization of rabbits and bovines	DNA vaccine	(94)
*msp5*			MSP	Characterization/immunization of mice	Titermax adjuvant	(95)
MSP2, MSP3, VirB9, &VirB10; OMP4, OMP9, Ef-Tu, Ana29, OMA87.	Dominant and subdominant antigens	R-proteins	MSP's, T4SS and other membrane proteins	Characterization/immune response	Saponin	(94)
rVirB9.1, rVirB9.2, rVirB10, rVirB11, and rEf-Tu	Dominant and subdominant antigens	R-proteins	T4SS	Characterization/immune response	Saponin	(40)
VirB2, VirB4-1, VirB4-2, VirB6-1, VirB7, VirB8-2, VirB9-1, VirB9-2, VirB10, VirB11, and VirD4	Dominant and subdominant antigens	R-proteins	T4SS/Synthetic overlapping peptides & recombinant proteins	Characterization/immune response	TiterMax Gold; silica vesicles, SV-100; Quil-A	(88, 96–98)
OmpA	Adhesin/invasin	R-proteins	Outer membrane protein	Characterization/Immune response	–	(23, 99, 100)
**Subdominant proteins**						
OMPs Am854 and Am779	Unknown function	Genomic study	OMP's	Conservation of candidate proteins	–	(101)
AM854, AM936	Mediate host cell invasion	R-proteins	OMP'S	Characterization/immune response	Saponin	(102)
AM779	Unknown function	R-proteins	OMP's	Characterization/immune response	Saponin	(103)
AM1108, AM127, and AM216	Unknown function/recombinant OMP's	R-proteins	OMP's	Characterization/immune response	–	(104)
Ana-29	Unknown function /recombinant OMP's	R-proteins	OMP's	Characterization/immune response	Quil-a; Quil A, Montanide ISA 50V and DEAE dextran mix	(105)
**Live, genetically modified organism**						
*A. marginale*	St. Maries- green-fluorescent protein expressing mutant	*In vitro* cultured	Live genetically modified organism	Characterization/immune response	Live agent	(106, 107)
*A. marginale*	Virginia-omp10:himar1 transposon mutant	*In vitro* cultured	Live genetically modified organism	Characterization/replication in bovines	Live agent	(108, 109)

List of some of the most important efforts to produce vaccines based on new approaches including DNA vaccines, recombinant antigens, synthetic peptides etc.

One of the first attempts to produce a recombinant DNA vaccine included the *msp1a* gene coupled to promoters of different origins into a vaccinia virus vector (84). After inoculating it into mice, this immunogen induced limited production of specific antibodies in experimental subjects.

Another DNA vaccine was constructed by fusing a sequence encoding B- and T-cell antigens from the *A. marginale msp1a* gene with a BVP22 domain and an invariant-chain MHC class II-targeting motif (fetal liver tyrosine kinase) capable of enhancing dendritic cell antigen uptake and presentation (85). This vaccine was inoculated in 6-month-old calves; it induced proliferative responses and expansion of gamma interferon-positive CD4^+^ T cells and immunoglobulin G responses against the linked B-cell epitope. However, no challenge with the live agent was reported.

A similar experiment used *msp1a, msp1b*, and *msp5* in the form of recombinant plasmids pET102-msp1α, pET101-msp1β, and pRSET-msp5. Inoculating a mixture of these plasmids primarily induced a Th2-type immune response in mice, and inoculating pET102-msp1α only induced an immunoglobulin response slightly higher than that of negative controls (86).

Using recombinant proteins is the next best choice. A mixture of rMSP1a, rMSP1b, rMSP4, and rMSP5 incorporated into ISCOM and ISCOMATRIX adjuvants induced the production of IgG1 and IgG2 in mice (86). A similar complex of the same recombinant MSPs stimulated the production of IgG, IgG1, and IgG2 when inoculated in mice (87, 93). Recombinant VirB2, VirB4-1, VirB4-2, VirB6-1, VirB7, VirB8-2, VirB9-1, VirB9-2, VirB10, VirB11, and VirD4 of the TFSS proteins were linked to major histocompatibility complex class II DRB3 antigens and were shown to induce IgG and stimulate CD4^+^ T cells from *A. marginale* membrane-immunized cattle. In these experiments, not all immunized animals responded to all TFSS proteins, yet most responded to recombinant VirB9-1, VirB9-2, and VirB10 both in antibody production and Th cell lines (96, 97). Inoculation of mice with recombinant VirB9-1 and VirB10 expressed in *Pichia pastoris* formulated with the self-adjuvanting silica vesicles, SV-100, and 200 μg of VirB9-1 and VirB10 induced higher antibody responses than a similar Quil-A saponin formulation (98). This same preparation induced a strong T-cell reaction in cells from calves previously immunized with *A. marginale* outer membranes (88).

Many studies used various options for immunizing mice, rabbits, or calves, from vector DNA vaccines to recombinant proteins with different adjuvants (Table 1). These studies include MSPs, TFSS proteins, outer membrane proteins, and other subdominant proteins, exemplifying many attempts for an immunoprophylactic solution to bovine anaplasmosis. Recombinant proteins have been tested in mice or calves for the type of immune response (Th1 or 2) (89, 110). In one case, steers immunized with a mixture of VirB9.1, VirB9.2, VirB10, VirB11, and EfTu produced the desired Th1-type immune response, but this response did not correlate with protection (40).

In addition to the “usual suspects,” other subdominant proteins with putative functions (at least in *A. marginale*) have been expressed in bacterial systems and used for immunization. Inoculation of AM854 (OmpA equivalent) and AM936 (Asp14 equivalent) incorporated with saponin induced both antigen-specific IgG1 and IgG2 in steers. Vaccinated animals developed higher levels of rickettsemia and greater packed cell volume (PCV) losses than controls immunized with *A. marginale* membrane fragments or negative controls when challenged with 10 *Anaplasma*–*Dermacentor andersoni*-infected ticks (102). In this study, *E. coli*-expressed recombinant antigens caused more severe bacteremia and clinical syndrome after a challenge. By contrast, the animals inoculated with membrane fractions, which included other proteins in addition to the native proteins of interest, did develop the desired Th-1 immune response.

Finally, a proof-of-concept study used an eight-branched multiple antigenic peptide (R10K-MAP) derived from Msp1a tandem repeat K;S as the antigen (90). The vaccination scheme comprised three doses (a prime boost-like scheme). The first dose consisted of a soluble inoculum applied subcutaneously on one side of the neck. The second dose was an ear implant that included (1) the antigen and one of two adjuvants, DEAE-dextran or Quil A (saponin), or (2) the same antigen with the two adjuvants. The third dose was either a second implant of the antigen with the same adjuvant or the antigen with the alternative adjuvant. After immunization, the experimental animals were challenged with a dose of 10^9^ recently thawed erythrocytes infected with a heterologous strain.

According to the clinical signs developed upon the challenge, the authors suggested that all (three) animals receiving a single adjuvant and one from each group receiving the two adjuvants were not protected from the disease (90). On the one hand, the results of this study are relatively consistent with the use of several adjuvants to increase the antigenicity of the chosen protein (105). On the other hand, this study only included three animals per group, so it is difficult to predict the effects on a larger group of animals. While initially promising, many questions should be answered before such a vaccine can be commercially released. For instance, what is the shortest time between implant application and exposure to a natural challenge in order to ensure adequate protection? Is this antigen the best option for immunization? Can other antigens be included in the implant? Finally, is the implant affordable?

#### Genetically modified organisms

Recombinant organisms are used for the immunoprophylaxis of many diseases (111). Transformation of *A. marginale* was only recently generated by transposon mutagenesis of the *A. marginale* Virginia strain. This mutant has extremely reduced expression of outer membrane protein (Omp)9, Omp8, Omp7, and Omp6 genes (108); it can be transmitted by ticks and shows reduced infectivity in both intact and splenectomized cattle (109). The authors did not test for protection against the Virginia wild type or a heterologous challenge. Another vaccine candidate was generated from a St. Maries strain with a transposon-mediated insertion of a 4.5-kb construct containing antibiotic resistance genes for selection and Turbo GFP as a marker. This strain, called AmStM-GFP, grows more slowly than the parent strain in culture (106). It induces immunity and similar clinical parameters for immunization with *A. centrale* but a lower maximum percentage of infected erythrocytes, a smaller drop in packed cell volume, and a longer time to reach peak bacteremia than wild-type AmStM (107). These new developments represent efforts to control the disease by means of live genetically modified organisms. However, their condition of live agents enables them to transmit other organisms like mycoplasmas and even viruses present in the culture systems.

## Perspectives and concluding remarks

More than a century has passed since Theiler (41) described both *A. marginale* and *A. centrale* as independent causal agents of bovine anaplasmosis and used the latter as a live vaccine. Until now, none of the options for vaccination against anaplasmosis prevent infection. Furthermore, vaccination is only expected to ameliorate the clinical signs should the animal get infected with *A. marginale* (42). This fact may be advantageous since, once an animal gets infected, it acquires a degree of permanent (concomitant) immunity that lasts throughout the animal's life span.

Since the early use of the *A. centrale* live vaccine, vaccination has not progressed beyond the use of an inactivated vaccine (in the United States). However, new advances include live attenuated *A. marginale*, recombinant proteins, synthetic peptides, genetically modified organisms, and an array of adjuvants and delivery systems like IL-2 and antigens linked to MHC class II molecules.

Vaccine design entails many obstacles, including, but is not restricted to, the induction of the desired immune response, the search for the ideal antigen(s) in terms of their contribution to virulence or metabolism, the expression of the antigens of interest at the key moment of pathogen development, and the feasibility of vaccine production, application, and commercialization. Furthermore, saponin has been the most widely used adjuvant for *A. marginale* vaccine production, followed by water-in-oil (w/o) and water-in-oil-in-water (w/o/w) emulsions, dextrans, and other commercially available mixtures. However, the results of the two trials mentioned previously showed that more than one adjuvant may be needed to stimulate several types of immune cells (90, 105).

In the case of recombinant or synthetic peptides or proteins, one should consider whether the protein is immunogenic and if the relevant epitopes are in the right conformation and within reach of the antibodies. Native proteins embedded within the membrane are immunogenic enough to induce protective immunity, but their recombinant counterparts might not be enough (22, 23). Furthermore, the fact that an antigen triggers the right immune response (IgG2) does not imply that the antigen is immunogenic enough to induce the same response alone or even that it is immunoprotective (40, 102). For example, subdominant proteins such as TFSS proteins appear as suitable vaccine candidates based on their location and function, but they did not induce immunoprotective immunity when tested.

The bleak panorama seems to indicate that we will never achieve cattle immunization against this formidable pathogen. However, the development of inactivated and mRNA vaccines against SARS-CoV-2 is a reminder of humanity's ability to confront health problems.

Live vaccines (*A. centrale*) have been an alternative for mass vaccination in several countries; vaccination with live cultivable GMOs may be a future alternative. New mutagenic techniques should be considered while designing live vaccines.

Furthermore, while it may seem that we are far from achieving the goal, the publication of more than 20 genome sequences of *A. marginale* at the NCBI should aid in the search for better vaccine candidates. Many outer membrane proteins and membrane-associated proteins have been tested as vaccine candidates and are yet to be developed into commercial vaccines. Small genomes of *A. marginale* comprise ~1,000 genes, with many of them (≥60%) still not described (8, 112). This fact should bring hope that other proteins involved in replication or metabolic or signaling pathways may be conserved among strains or may perform vital functions for the parasite survival, providing antigen candidates. New forms of antigen delivery (microtubules, nanoparticles, MAP, etc.) (88, 89, 98, 110) should also be tested to provide promising antigens with the best chance to induce the desired immune response. Finally, the experience of live and whole-cell immunogens suggests that a single antigen hardly induces the appropriate immune response or protects against deliberate or natural challenges. Thus, vaccination experiments with recombinant or synthetic proteins/peptides should include many candidates to mimic the immune response of the whole-cell inactivated or live vaccines.

Thus, we expect bovine anaplasmosis immunization to be realized in the near future, contributing to safer and more profitable cattle production.

## Author contributions

SR: conceptualization, review, and editing. ES-E: design of Figure 1. SR, ES-E, MC-C, IA-E, and JP: investigation and original draft preparation and writing. All authors have read and agreed to the published version of the manuscript.

## Funding

This work was supported by the INIFAP project (SIGI Number 11231734788).

## Conflict of interest

The authors declare that the research was conducted in the absence of any commercial or financial relationships that could be construed as a potential conflict of interest.

## Publisher's note

All claims expressed in this article are solely those of the authors and do not necessarily represent those of their affiliated organizations, or those of the publisher, the editors and the reviewers. Any product that may be evaluated in this article, or claim that may be made by its manufacturer, is not guaranteed or endorsed by the publisher.
